# Glucose Loading Enhances the Value of ^18^F-FDG PET/CT for the Characterization and Delineation of Cerebral Gliomas

**DOI:** 10.3390/cancers12071977

**Published:** 2020-07-20

**Authors:** Dongwoo Kim, Hae Young Ko, Sangwon Lee, Yong-ho Lee, Sujin Ryu, Seon Yoo Kim, Jee-in Chung, Misu Lee, Ju Hyung Moon, Jong Hee Chang, Mijin Yun

**Affiliations:** 1 Department of Nuclear Medicine, Severance Hospital, Yonsei University College of Medicine, Seoul 03722, Korea; KDWOO@yuhs.ac (D.K.); HYKO23@yuhs.ac (H.Y.K.); LSW0423@yuhs.ac (S.L.); SYKIM1703@yuhs.ac (S.Y.K.); JICHUNG@yuhs.ac (J.-i.C.); 2 Department of Internal Medicine, Severance Hospital, Yonsei University College of Medicine, Seoul 03722, Korea; YHOLEE@yuhs.ac; 3 Brain Tumor Center, Severance Hospital, Yonsei University Health System, Seoul 03722, Korea; ITNWLS@yuhs.ac; 4 Division of Life Sciences, College of Life Science and Bioengineering, Incheon National University, Incheon 22012, Korea; misulee@inu.ac.kr; 5 Department of Neurosurgery, Brain Tumor Center, Severance Hospital, Yonsei University College of Medicine, Seoul 03722, Korea; MJJR80@yuhs.ac (J.H.M.); CHANGJH@yuhs.ac (J.H.C.)

**Keywords:** glioma, glucose loading, ^18^F-FDG PET/CT, grading

## Abstract

This study aimed to assess how to enhance the value of ^18^F-Fluorodeoxyglucose (FDG) PET/CTs for glioma grading and better delineation of the tumor boundary by glucose loading. In mouse models of brain tumor using U87MG cells, ^18^F-FDG-PET images were obtained after fasting and after glucose loading. There was a significant difference in the tumor-to-normal cortex-uptake ratio (TNR) between the fasting and glucose-loading scans. ^14^C-2-Deoxy-D-glucose (^14^C-DG) uptake was measured in vitro using U87MG, U373MG and primary neurons cultured with different concentrations of glucose. The tumor-to-neuron ratio of ^14^C-DG uptake increased with up to 10 mM of glucose. Finally, 10 low-grade and 17 high-grade glioma patients underwent fasting and glucose loading ^18^F-FDG PET/CT and the TNR was compared between scans. The effect of glucose loading was significant in high-grade but not in low-grade gliomas. The receiver operating characteristic curve analyses with a cut-off TNR of 0.81 showed a higher area under the curve after glucose loading than fasting for differentiating low-grade versus high-grade gliomas. In addition, the glucose loading PET/CT was more useful than the fasting PET/CT for the discrimination of oligodendrogliomas from IDH-wildtype glioblastomas. Glucose loading resulted in a greater reduction in ^18^F-FDG uptake in the normal cortex than in tumors, which increases the usefulness of ^18^F-FDG PET/CT for grading.

## 1. Introduction

^18^F- Fluorodeoxyglucose (FDG) PET/CTs provide information based on the increased glycolysis of cancer cells, and are successfully used for the diagnosis, staging, treatment response monitoring, and prognostication of cancers. In cerebral gliomas, higher ^18^F-FDG uptake correlates with higher pathological grades and patient prognosis [1]. Although ^18^F-FDG PET/CT is accessible and validated for functional tumor imaging, it has limitations for cerebral gliomas. Most significantly, there is a high rate of physiologic glucose uptake in the cerebral cortex, which can be as high as high-grade tumors or higher than low-grade gliomas [2,3]. Therefore, accurate characterization of the tumor and delineation of the tumor boundary is challenging with ^18^F-FDG PET/CT.

Radiolabeled amino acid tracers, which are associated with increased cellular proliferation and protein synthesis are considered superior alternatives to ^18^F-FDG for cerebral gliomas. They are excellent for tumor detection and delineation due to their low background uptake in the normal cortex and high radiolabeled amino acid uptake in tumors, including low-grade gliomas [4]. However, the predictive values for grading are less clear because of the significant overlap in amino acid metabolism between grades [5]. In particular, the ability to discriminate oligodendrogliomas (ODs) from high-grade astrocytic gliomas is challenging with radiolabeled amino acid tracers. Magnetic resonance imaging (MRI) has been widely used as a diagnostic modality for gliomas. However, enhanced MRI has limitations in that it cannot accurately provide the stage of the glioma or discriminate clearly between the tumor and the surrounding edema [6,7]. Recently, advanced MRI techniques, have been used to provide functional, physiological and molecular information on glioma, including perfusion MRI using relative cerebral blood volume (rCBV) and magnetic resonance spectroscopy (MRS) using the concentration of water-soluble metabolites. However, MRS is not able to provide a clear image due to the presence of susceptibility artefacts and it has methodological limitations due to the lack of standardization for image acquisition and processing [8].

The results of different studies vary with regard to the correlation between the blood glucose level and ^18^F-FDG uptake in different organs and tumors. However, all studies agree that there is a significant inverse relationship between blood glucose level and ^18^F-FDG uptake in the normal cerebral cortex [9,10,11,12]. A blood glucose level above 110 mg/dL decreases ^18^F-FDG uptake in the cerebral cortex [12]. The guidelines for tumor imaging recommend at least 4 h of fasting before ^18^F-FDG PET/CT to avoid reducing the tumor detection sensitivity [13,14]. However, for cerebral gliomas, an increased blood glucose level might be beneficial because it decreases the background ^18^F-FDG uptake in the cerebral cortex, and thus ensures high tumor-to-normal cortex contrast. In this study, the effect of various blood glucose levels on ^18^F-FDG uptake in the normal cerebral cortex and cerebral gliomas, and the blood glucose level required to obtain the best TNR were investigated in mouse tumor models, glioblastoma (GBM) cell lines, and patients with cerebral gliomas.

## 2. Results

### 2.1. TNR of ^18^F-FDG Uptake Was Increased by Glucose Loading in a Mouse GBM Model

**Figure 1:**
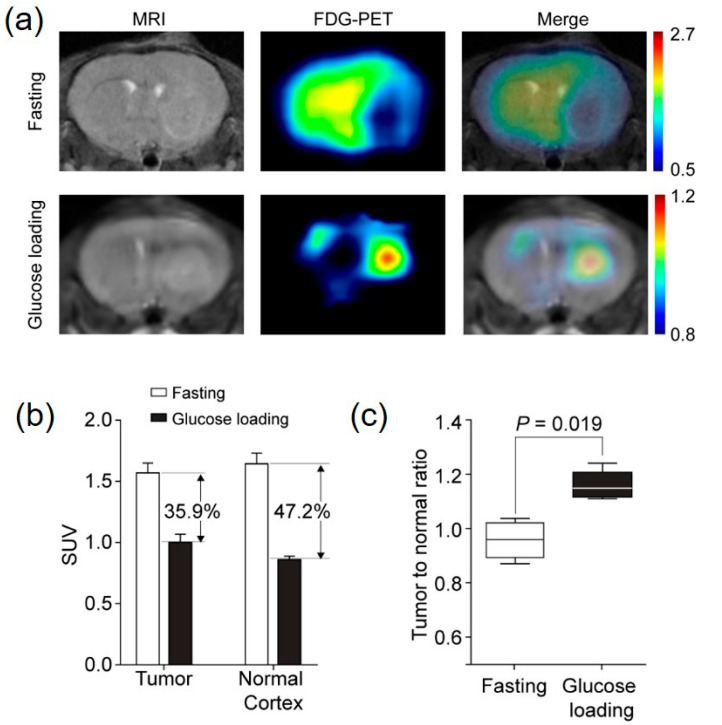
(**a**) ^18^F-FDG PET/MRI images of mice U87MG xenograft models after fasting and with glucose loading. (**b**) Maximum standardized uptake value (SUVmax) in the tumor and normal cortex after fasting and glucose loading. (**c**) Tumor to contralateral cortex ratio after fasting and glucose loading.

At the time of the ^18^F-FDG injection, the mean blood glucose level of mice was 74.66 ± 7.42 mg/dL after fasting and 122.53 ± 21.19 mg/dL after glucose loading. The tumor was difficult to delineate visually on the ^18^F-FDG/PET images due to the high ^18^F-FDG uptake in the normal cortex after fasting. On the contrary, after glucose loading, ^18^F-FDG uptake in the normal cortex decreased significantly, which resulted in clear delineation of the tumor on the PET images (Figure 1a). The decreased rate in the standardized uptake value (SUV) after glucose loading was 35.9% in the tumor (SUVmax) and 47.2% in the normal cortex (SUVmean) (Figure 1b). There was a statistically significant difference in the TNR between the fasting and glucose loading scans (0.95 vs. 1.15, *p* = 0.019) (Figure 1c).

### 2.2. ^14^C-DG Uptake Ratio of GBM Cells to Neurons Was Enhanced at High Glucose Levels

**Figure 2:**
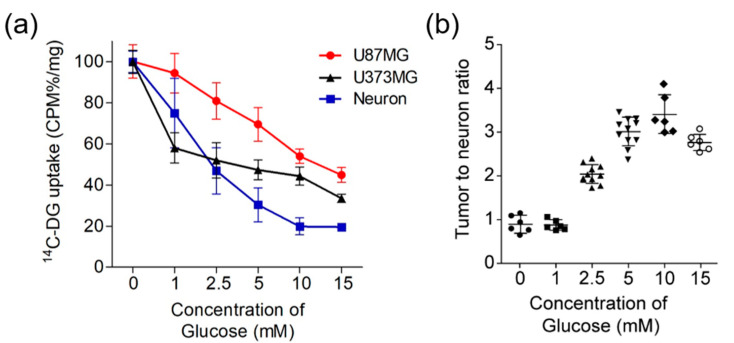
In vitro ^14^C-2-Deoxy-D-glucose (^14^C-DG) uptake in U87MG, U373MG and mouse primary neurons. (**a**) ^14^C-DG uptake and (**b**) U87MG tumor-to-neuron ratio for different glucose concentrations in the media.

For in vitro cell uptake, we replaced positron-emitting nuclide ^18^F-FDG with beta-decay radionuclide ^14^C-DG, which has the same structure and function, but is easier to use in vitro. We screened ^14^C-DG uptake in mouse primary neurons, astrocytes, U87MG and U373MG cells and found that the ^14^C-DG uptake in astrocytes was not significantly different regardless of the glucose concentration in the media. Therefore, we evaluated changes in ^14^C-DG uptake with different glucose levels in the media-only in the mouse primary neurons, U373MG and U87MG cells. The concentration of cold glucose in the media was varied at 0, 1, 2.5, 5, 10, and 15 mM. ^14^C-DG uptake was highest at 0 mM of glucose in all cell types (U87MG, 3.11 ± 0.81, CPM%/mg; U373MG, 2.89 ± 0.41; neuron, 3.54 ± 0.37, CPM%/mg). While ^14^C-DG uptake decreased as the glucose concentration in the media increased, there were differences in the rates of decrease among the primary neurons, U87MG and U373MG cells (Figure 2a). The U87MG tumor-to-neuron ratio of ^14^C-DG uptake increased continuously up to 10 mM of glucose concentration (1.00 at 0 mM; 1.25 at 1 mM; 1.72 at 2.5 mM; 2.11 at 5 mM; 2.74 at 10 mM), and then decreased to 2.29 at 15 mM (Figure 2b). The U373MG tumor-to-neuron ratio of ^14^C-DG uptake (1.00 at 0 mM; 0.77 at 1 mM; 1.11 at 2.5 mM; 1.56 at 5 mM; 2.23 at 10 mM; 1.71 at 15 mM) had a similar pattern toU87MG.

### 2.3. TNR of ^18^F-FDG PET/CT Images Was Increased by Glucose Loading in Patients with High-Grade Gliomas

**Figure 3:**
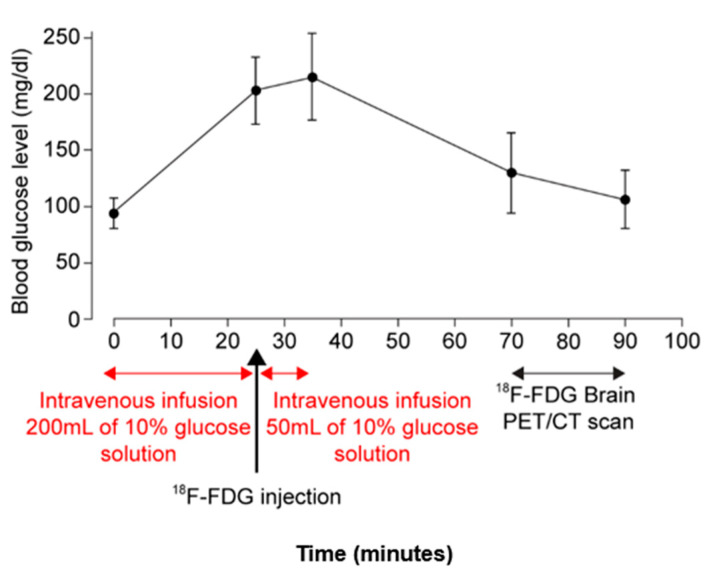
Blood glucose level by intravenous infusion of 10% glucose solution.

The clinicopathologic characteristics of the patients are summarized in Table 1. Of the 27 patients with histologically confirmed cerebral gliomas, 10 (37.0%) had WHO grade II tumors, 7 (25.9%) had grade III tumors, and 10 (37.0%) had grade IV tumors. The mean blood glucose level of the 27 patients at the time of ^18^F-FDG injection was 98.9 mg/dL after fasting and 203.1 mg/dL after glucose loading. In the glucose loading study, the changes in blood glucose levels over the course of the study are shown in Figure 3.

**Figure 4:**
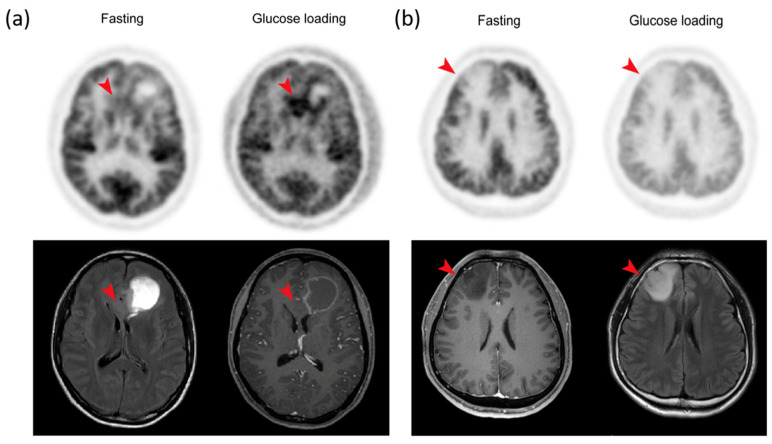
^18^F-FDG PET/CT (upper panel) and FLAIR and T1 enhanced MRI images (bottom panel) after fasting and glucose loading in a patient with (**a**) high-grade and (**b**) low-grade glioma. (**a**) Involvement of glioblastoma was pathologically confirmed in the left frontal lobe and corpus callosum (red arrow) in the glioblastoma. (**b**) Diffuse astrocytoma, IDH-mutant was pathologically confirmed in the right frontal lobe (red arrow).

In all patients, the median SUVmax of tumors was 8.84 (interquartile range (IQR), 7.27–10.38) after fasting and 5.13 (IQR, 3.52–5.87) after glucose loading. The median SUVmean of the contralateral normal cortex was 9.75 (8.47–11.17) after fasting and 4.70 (3.23–5.34) after glucose loading. In the case of high-grade glioma, glucose loading induced a significant reduction in ^18^F-FDG uptake in the tumor, and more in the normal cortex (42.5% in tumors versus 53.6% in the normal cortex, *p* = 0.0261) (Figure 4a).

When the tumors were categorized as high (Grade III and IV, *n* = 17) and low-grade (Grade II, *n* = 10) gliomas, the effect of glucose loading was different (Figure 4a,b). In high-grade gliomas, the median TNR was 0.96 (IQR, 0.78–1.07) after fasting and 1.20 (IQR, 1.01–1.28) after glucose loading (increase of 25%, *p* = 0.0167). However, in low-grade gliomas, the median TNR was 0.66 (IQR, 0.52–0.77) after fasting and 0.68 (IQR, 0.55–0.76) after glucose loading (*p* = 0.7055) (Table 2). With a cut-off TNR of 0.81 for differentiating low-grade versus high-grade gliomas, the receiver operating characteristic (ROC) analyses showed a higher area under the curve (AUC) after glucose loading than that after fasting (0.953 vs. 0.847; *p* = 0.0431).

**Figure 5:**
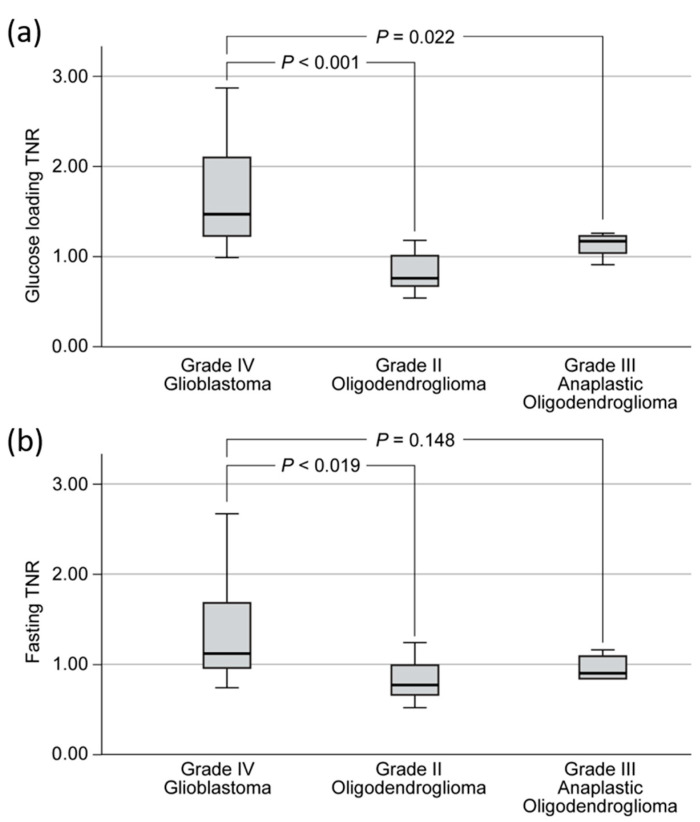
^18^F-FDG TNRs of grade II oligodendrogliomas, grade III anaplastic oligodendrogliomas, and grade IV IDH-wildtype glioblastomas in (**a**) glucose loading ^18^F-FDG PET/CT and (**b**) fasting ^18^F-FDG PET/CT.

Glucose loading PET/CT was more useful than fasting PET/CT for the discrimination of ODs from IDH-wildtype GBMs. GBMs showed higher TNRs (median, 1.71; IQR, 1.28–2.19) than Grade II ODs (median, 0.76; IQR, 0.65–1.05; *p* < 0.001, adjusted) and Grade III ODs (median, 1.17; IQR, 1.01–1.24; *p* = 0.022, adjusted) in glucose loading PET/CT (Figure 5a). In contrast, the TNRs differed significantly between IDH-wildtype GBMs (median, 1.58; IQR, 1.03–1.70) and Grade II ODs (median, 0.77; IQR, 0.64–1.05; *p* = 0.019, adjusted), but there was no significant difference in TNRs between IDH-wildtype GBMs and Grade III ODs (median, 0.90; IQR, 0.84–1.11; *p* = 0.148, adjusted) after fasting PET/CT (Figure 5b).

## 3. Discussion

A high blood glucose level decreases radioactive glucose uptake in tumors. Therefore, at least 4 h of fasting is recommended so as not to decrease detection sensitivity before an ^18^F-FDG PET/CT in patients with cerebral gliomas [13,14]. However, fasting increases background ^18^F-FDG uptake in the normal cortex. On the other hand, high blood glucose levels decrease ^18^F-FDG uptake in the normal cortex, with the risk of decreasing ^18^F-FDG uptake in the tumors. In this prospective study, we found that glucose loading led to a greater reduction in ^18^F-FDG uptake in the normal cortex than in tumors, especially in high-grade gliomas. Thus, the TNR increased after glucose loading, which increased the usefulness of ^18^F-FDG PET/CT for grading cerebral gliomas.

Orthotopic mouse tumor models using the human GBM cell line (U87MG) were imaged twice with ^18^F-FDG PET (once with fasting and once with glucose loading) to determine the feasibility of glucose loading for enhancing tumor to normal cortex contrast. After fasting, ^18^F-FDG uptake in the normal cerebral cortex was higher than in the tumor. In fact, the tumor was shown as a round, cold defect compared to the cortex, and the delineation of the tumor for the SUV measurement was difficult without MRI co-registration. In contrast, ^18^F-FDG uptake in the normal cortex decreased significantly, and there was a clear delineation of the tumor on PET/CT images after glucose loading. The tumor appeared clear, and the SUV measurement could be performed without the help of MRI co-registration. The higher reduction (49.2%) in the SUVmax in the normal cortex compared to that (35.9%) in the tumor led to a significantly higher TNR after glucose loading. The mouse tumor models showed the potential benefits of glucose loading in terms of increasing the contrast between the tumor and the normal cortex.

Studies have evaluated the effect of high blood glucose levels on ^18^F-FDG uptake in the cerebral cortex, liver, and blood pool. The normal cerebral cortex is the organ that is most consistently affected by different levels of blood glucose. In a recent report, the mean brain SUVmax reduced gradually with a blood glucose level of 110 mg/dL or higher. The SUVmax of the cerebral cortex was reduced by 20, 35, 50, 60, and 65% for blood glucose ranges of 111–120, 121–140, 141–160, 161–200, and greater than 20 mg/dL, respectively [9]. This earlier study suggested a formula to correct SUV in which the measured SUVmax was multiplied by a reduction factor of 1.25, 1.5, 2, 2.5, and 2.8 for the same blood glucose ranges. Regardless, there is limited information on the effect of various blood glucose levels on ^18^F-FDG uptake in cerebral gliomas and the blood glucose level, and which level can obtain the highest TNR. Using the U87MG, U373MG cell lines and primary neuron with different concentrations of glucose ranging from 0 to 15 mM, we found decreased ^14^C-DG uptake at all glucose levels in all three types of cells, but the rates of decrease differed among the primary neurons, U87MG and U373MG cells. ^14^C-DG uptake of U87MG and U373MG cells showed similar values to neurons at low concentrations of glucose, but higher values than neurons at high concentrations of glucose. The tumor-to-neuron ratio increased continuously up to 10 mM and then decreased from 15 mM. Glucose uptake remained higher in the tumor cells than the primary neurons even at higher concentrations of glucose.

So far, only one report has evaluated the effect of high blood glucose levels on brain tumor detection. Ishizu et al. showed that glucose loading enhanced the detection of brain tumors in eight patients with recurrent or residual glioma and one patient with brain metastasis [15]. They started the infusion of 10% glucose solution 10–15 min before the ^18^F-FDG injection, and maintained it until the end of the study, finally providing approximately 500 mL (50 g of glucose). The glucose loading decreased ^18^F-FDG uptake in the cerebral cortex (54.2% ± 13.8%) in an almost inverse proportion to the glucose level while the tumors also showed a decrease (42.5% ± 15.6%), and the resulting TNR increased by 26.0% ± 5.7%. Although these results are feasible, the limited number of patients included in the study (6 GBMs, 1 OD, 1 astrocytoma) make it difficult to accurately evaluate the value of glucose loading.

In this study, based on the results of the previous report and our cell line and mouse studies, we performed ^18^F-FDG PET/CT scans after fasting and intravenous infusion of 250 mL of 10% glucose for a total of 35 min, 25 min before ^18^F-FDG injection and 10 min after the injection. Glucose loading induced a significant reduction in ^18^F-FDG uptake in both the tumor and normal cortex (42.5% in tumors vs. 53.6% in the normal cortex, *p* = 0.0261). The effect of high blood glucose was more beneficial in high-grade than low-grade gliomas. In high-grade gliomas, the median TNR increased by 25% after glucose loading compared to fasting. In low-grade gliomas, there were no significant differences in the median TNRs between fasting and glucose loading. Previously, fasting FDG PET/CT studies have shown relatively low AUC values for grading high- vs. low-grade gliomas (AUC: 0.76–0.80) [15,16]. MRI studies have also shown similar AUC values (0.78–0.86) [17,18]. In our study, with a TNR cut-off of 0.81, we found a significantly higher AUC value for grading after glucose loading than fasting (0.953 vs. 0.847; *p* = 0.0431). This high diagnostic power could help patients who cannot undergo any surgical procedures or biopsy due to the location of lesions. Further studies are needed to see whether additional data from advanced MRI techniques, including perfusion MRI, apparent diffusion coefficient (ADC), and MRS will improve the performance of glucose loading ^18^F-FDG PET/CT.

Currently, radiolabeled amino acid tracers such as ^11^C-methionine, 3,4-dihydroxy-6-^18^F-fluoro-l-phenylalanine (^18^F-FDOPA), and O-(2-^18^F-fluoroethyl)-L-tyrosine (^18^F-FET) are preferred to ^18^F-FDG. Other than lower background uptake in the cortex, they are better at showing IDH-wildtype low-grade gliomas [16,17,18]. However, their value in differentiating low-grade vs. high-grade glioma needs further validation due to the high uptake in ODs, which is as high as that for high grade IDH1-wildtype gliomas [4]. In this study, glucose loading ^18^F-FDG PET/CT was able to discriminate ODs from IDH-wildtype GBMs. Although high and variable normal cortical uptake has limited the use of ^18^F-FDG PET/CT, ^18^F-FDG has advantages in representing the metabolic hallmarks of malignant tumors. Based on our study, ^18^F-FDG PET/CT with glucose loading might be an opportunity to revisit the use of ^18^F-FDG in patients with cerebral gliomas. Additional studies comparing ^18^F-FDG PET/CT with glucose loading and amino acid radiotracers might help to further evaluate this issue.

There were limitations to this study. Patients with diabetes were not included because their baseline blood glucose levels can be highly variable. Further studies are needed to personalize glucose loading methods based on a patient’s individual diabetic status and to control excessive hyperglycemia. Secondly, the metabolic tumor volume related to patient prognosis was not measured, although it was easier to delineate tumor margins due to the lower background in high-grade tumors. Future studies are needed to investigate whether ^18^F-FDG PET/CT after glucose loading makes it easier to determine the resection margin and metabolic tumor volume of high-grade cerebral gliomas.

## 4. Materials and Methods

### 4.1. Glioblastoma Mouse Model

All animal experiments were approved by the Institutional Animal Care and Use Committee (IACUC) of Yonsei University College of Medicine (approval code 2018-0202). Balb/c nude female mice 6 to 8 weeks old (OrientBio, Seongnam, Korea) were used for all animal experiments. U87MG (human glioblastoma) cells (5 × 10^5^ in 2 μL phosphate buffered saline [PBS]) were stereotactically implanted into the right frontal lobe of the mice. The glioma-bearing mice were imaged 4 weeks post-implantation.

### 4.2. Animal PET

All mice (*n* = 5) were imaged twice on a microPET scanner (Inveon, Siemens Healthcare, Erlangen, Germany), once after overnight fasting and again after glucose loading. For ^18^F-FDG PET imaging, the mice were anesthetized with 2.5% isoflurane. In the glucose loading study, blood glucose levels were measured using a glucometer (Accu-Chek Performa, Accu-Chek Inform II test strips; Roche Diagnostics, Indianapolis, IN, USA) 5 min after the subcutaneous injection of 5% dextrose solution (200 μL). Then, the mice were intravenously administered with ^18^F-FDG (7.4 MBq/0.2 mL in saline) and allowed to rest for 1 h on a heating pad under 2% isoflurane anesthesia. ^18^F-FDG PET data were acquired for 10 min. After imaging, the animals were kept under a heat lamp until awake, then placed in an isolation room for 24 h to eliminate radiation hazards. The PET data were reconstructed with three-dimensional (3D) ordered subset expectation-maximization (OSEM) with 2 iterations and 18 subsets. The matrix size was 128 × 128 × 159, and the voxel size was 0.776 × 0.776 × 0.796 mm. The SUV was calculated by the tissue concentration kBq/cm^3^ per injected dose (kBq per gram of body weight).

### 4.3. Animal Magnetic Resonance Imaging

Magnetic resonance imaging (MRI) of the implanted brain tumor was performed using a 9.4 T preclinical MRI (Bruker BioSpec 94/20 USR, Ettlingen, Germany; software: ParaVision 5.0) with water-cooled gradient coils (maximum gradient strength, 400 mT/m) before PET imaging. The mice were kept warm on a heating pad and anesthetized by 2% isoflurane during the MRI, which was performed with a respiration-triggered and fat-suppressed T2-weighted rapid acquisition with relaxation enhancement (RARE) sequence [19]. A two-dimensional (2D) set of axial images were acquired (TR, 4900 ms; TEeff, 24 ms; pixel-size, 208 μm^2^; slice-thickness, 500 μm).

### 4.4. Animal Image Analysis

Image processing and analyses were performed using PMOD software (PMOD Technologies Ltd., Zurich, Switzerland). ^18^F-FDG PET images were co-registered to the corresponding T2-weighted MRI images and manually transformed for minor misalignments. Two regions of interest (ROI) were manually drawn over the MRI images (one for the tumor to measure the maximum SUV and the other for the contralateral cortex to measure the mean SUV). The TNR were calculated and the fasting and glucose loading scans were compared.

### 4.5. In Vitro ^14^C-DG Uptake in Glioma Cells and Neurons

The cells (U87MG, U373MG and mouse primary neuron: 5 × 10^4^) were seeded in 1 mL growth medium per well in 24-well plates and incubated in a standard culture medium for 24 h. The cells were washed twice with glucose-free medium and cultured in 0, 1, 2.5, 5, 10, and 15 mM glucose-concentration medium for 2 h. Subsequently, ^14^C–DG (PerkinElmer, Waltham, MA, USA) 3.7 kBq/mL was added to each sample, and the cells were incubated for 30 min. Then, the medium was removed and the cells were rinsed twice with ice-cold PBS. The resultant cells were lysed with 500 μL of 0.2 N NaOH for 2 h at room temperature. The radioactivity was measured with a liquid scintillation counter (Tri-Carb, PerkinElmer, Waltham, MA, USA). The measured radioactivity was normalized to protein concentration by previously described methods [20]. The experiments were performed three times using three replicates for each condition.

### 4.6. Patients

This prospective study included 27 patients suspected of cerebral gliomas (11 men, 16 women; median age, 50.0 years; range, 27–75 years) between June 2019 and December 2019. All patients had undergone contrast-enhanced MRI (CE-MRI) and two ^18^F-FDG PET/CT scans, one after fasting and the other after glucose loading. The interval between the two ^18^F-FDG PET/CT scans was 1 to 14 days, with a median interval of 1 day. Patients underwent surgery shortly after imaging (median, 3 days; range, 1–20 days). No therapeutic intervention, such as any other surgery, radiotherapy, or chemotherapy was performed before surgery in any of the patients. All gliomas were classified using the 2016 World Health Organization (WHO) guidelines. The Institutional Review Board of Yonsei University College of Medicine approved this prospective study (approved code: 2018-0202), and informed consent was obtained from all patients (IRB No. 4-2019-0455). This study was registered at cris.nih.go.kr (KCT0004466).

### 4.7. ^18^F-FDG PET/CT Protocol

For fasting scans, all patients fasted for at least 6 h, then, blood glucose concentration was confirmed as less than 140 mg/dL before an injection of ^18^F-FDG. For glucose loading scans, all patients were asked to fast for at least 6 h to ensure their blood glucose level was less than 140 mg/dL. The glucose loading was started by intravenous infusion of 250 mL of 10% glucose solution, with 200 mL given first for 25 min at a rate of 8 mL/min, then, approximately 5.5 MBq of ^18^F-FDG per kilogram of body weight was administered, and finally, the remaining 50 mL of 10% glucose solution was further infused for 10 min (infusion speed of 5 mL/min). Blood glucose levels were measured five times: on arrival, just before ^18^F-FDG injection, 10 min after ^18^F-FDG injection, before scanning, and after scanning.

Imaging was performed using a PET/CT scanner (Discovery 600, GE Healthcare, Waukesha, WI, USA) with an axial field-of-view of 15.7 cm and a spatial resolution of 4.0 mm at full-width at half maximum at 1 cm from center. Sixty minutes after the ^18^F-FDG injection, CT images were obtained with an eight-slice helical CT unit (Light Speed, GE Healthcare, Waukesha, WI, USA) using the following parameters: 120 kVp, 200 mA; 0.8 s rotation time; 3.3 mm scan reconstruction; 25 cm field-of-view; and a 256 × 256 matrix. Whole-brain, 3D PET images were obtained over 10 min. PET data were reconstructed iteratively using an ordered-subset expectation-maximization algorithm. The CT datasets were used for attenuation correction.

### 4.8. Data Analysis

PET/CT images were reviewed and analyzed on a dedicated workstation by two nuclear medicine physicians who reached consensus. The PET/CT images were registered to contrast-enhanced MRI images using a fusion software (MIM-6.5; MIM Software Inc., Cleveland, OH, USA). A volume of interest (VOI) was manually drawn over the hottest area of each tumor, and the SUVmax of the tumor was measured. When no increases in ^18^F-FDG uptake were observed, the SUVmax was measured within the boundary of the tumor on the MRI images. The SUV of a VOI was calculated as follows: (decay-corrected activity (kBq per tissue volume (mL))/(injected ^18^F-FDG activity (kBq) per body mass (g)). To obtain reference (background) regions, three VOIs were manually drawn in the regions of the contralateral cerebral cortex with a normal appearance, the SUVmean values of the three VOIs were averaged, and the TNR was calculated.

### 4.9. Statistical Analysis

The Mann–Whitney *U* test was used to compare the TNR of the ^18^F-FDG uptake on PET after fasting versus after glucose loading. ROC analysis was used to compare the diagnostic performance of the two ^18^F-FDG PET/CT protocols for glioma grading. The Bonferroni methods was used in post hoc analyses. All statistical analyses were performed using IBM SPSS Statistics for Windows, version 25 (IBM Corp, Armonk, NY, USA). Except for post hoc analyses, a *p*-value less than 0.05 was considered statistically significant.

## 5. Conclusions

Compared to fasting, glucose loading resulted in a higher tumor to normal cortex contrast in high-grade gliomas, and subsequently, improved glioma grading and the discrimination of oligodendrogliomas from IDH-wildtype glioblastomas.

## Figures and Tables

**Table 1:** Patient characteristics.

Characteristic	Values
Age (y)	Median, 50 (range, 27–75)
Sex, *n* (%)	
Male	11 (40.7%)
Female	16 (59.3%)
WHO 2016 grade, *n* (%)	
Grade II	10 (37.0%)
Grade III	7 (25.9%)
Grade IV	10 (37.0%)
Histology, *n* (IDH-mutant/wildtype)	
Astrocytoma	20
Grade II	6 (5/1)
Grade III	4 (1/3)
Grade IV	10 (9/1)
Oligodendroglioma	7
Grade II	4
Grade III	3

WHO, World Health Organization.

**Table 2:** The tumor-to-normal brain cortex uptake ratio (TNR) in glioma.

State	High-Grade Median (IQR) (*n* = 17)	Low-Grade Median (IQR) (*n* = 10)
Glucose loading	1.20 (IQR 1.01–1.28)	0.68 (IQR 0.55–0.76)
Fast	0.96 (IQR 0.78–1.07)	0.66 (IQR 0.52–0.77)
(*p* value)	0.0167	0.7055

## References

[1] Kim D., Kim S., Kim S.H., Chang J.H., Yun M. (2018). Prediction of Overall Survival Based on Isocitrate Dehydrogenase 1 Mutation and 18F-FDG Uptake on PET/CT in Patients With Cerebral Gliomas. Clin. Nucl. Med..

[2] Kato T., Shinoda J., Nakayama N., Miwa K., Okumura A., Yano H., Yoshimura S., Maruyama T., Muragaki Y., Iwama T. (2008). Metabolic assessment of gliomas using 11C-methionine, [18F] fluorodeoxyglucose, and 11C-choline positron-emission tomography. Am. J. Neuroradiol..

[3] Chung J.K., Kim Y.K., Kim S.K., Lee Y.J., Paek S., Yeo J.S., Jeong J.M., Lee D.S., Jung H.W., Lee M.C. (2002). Usefulness of 11C-methionine PET in the evaluation of brain lesions that are hypo- or isometabolic on 18F-FDG PET. Eur. J. Nucl. Med. Mol. Imaging.

[4] Kim D., Chun J.H., Kim S.H., Moon J.H., Kang S.G., Chang J.H., Yun M. (2019). Re-evaluation of the diagnostic performance of (11) C-methionine PET/CT according to the 2016 WHO classification of cerebral gliomas. Eur. J. Nucl. Med. Mol. Imaging.

[5] Singhal T., Narayanan T.K., Jain V., Mukherjee J., Mantil J. (2008). 11C-L-methionine positron emission tomography in the clinical management of cerebral gliomas. Mol. Imaging Biol..

[6] Bangiyev L., Rossi Espagnet M.C., Young R., Shepherd T., Knopp E., Friedman K., Boada F., Fatterpekar G.M. (2014). Adult brain tumor imaging: State of the art. Semin. Roentgenol..

[7] Jansen E.P., Dewit L.G., van Herk M., Bartelink H. (2000). Target volumes in radiotherapy for high-grade malignant glioma of the brain. Radiother. Oncol..

[8] Langen K.J., Galldiks N., Hattingen E., Shah N.J. (2017). Advances in neuro-oncology imaging. Nat. Rev. Neurol..

[9] Sprinz C., Altmayer S., Zanon M., Watte G., Irion K., Marchiori E., Hochhegger B. (2018). Effects of blood glucose level on 18F-FDG uptake for PET/CT in normal organs: A systematic review. PLoS ONE.

[10] Sprinz C., Zanon M., Altmayer S., Watte G., Irion K., Marchiori E., Hochhegger B. (2018). Effects of blood glucose level on 18F fluorodeoxyglucose (18F-FDG) uptake for PET/CT in normal organs: An analysis on 5623 patients. Sci. Rep..

[11] Eskian M., Alavi A., Khorasanizadeh M., Viglianti B.L., Jacobsson H., Barwick T.D., Meysamie A., Yi S.K., Iwano S., Bybel B. (2019). Effect of blood glucose level on standardized uptake value (SUV) in (18)F- FDG PET-scan: A systematic review and meta-analysis of 20,807 individual SUV measurements. Eur. J. Nucl. Med. Mol. Imaging.

[12] Sarikaya I., Sarikaya A., Sharma P. (2019). Assessing effect of various blood glucose levels on (18)F-FDG activity in the brain, liver and blood pool. J. Nucl. Med. Technol..

[13] Delbeke D., Coleman R.E., Guiberteau M.J., Brown M.L., Royal H.D., Siegel B.A., Townsend D.W., Berland L.L., Parker J.A., Hubner K. (2006). Procedure guideline for tumor imaging with 18F-FDG PET/CT 1.0. J. Nucl. Med..

[14] Boellaard R., Delgado-Bolton R., Oyen W.J., Giammarile F., Tatsch K., Eschner W., Verzijlbergen F.J., Barrington S.F., Pike L.C., Weber W.A. (2015). FDG PET/CT: EANM procedure guidelines for tumour imaging: Version 2.0. Eur. J. Nucl. Med. Mol. Imaging.

[15] Ishizu K., Nishizawa S., Yonekura Y., Sadato N., Magata Y., Tamaki N., Tsuchida T., Okazawa H., Miyatake S., Ishikawa M. (1994). Effects of hyperglycemia on FDG uptake in human brain and glioma. J. Nucl. Med..

[16] Lopci E., Riva M., Olivari L., Raneri F., Soffietti R., Piccardo A., Bizzi A., Navarria P., Ascolese A.M., Ruda R. (2017). Prognostic value of molecular and imaging biomarkers in patients with supratentorial glioma. Eur. J. Nucl. Med. Mol. Imaging.

[17] Fueger B.J., Czernin J., Hildebrandt I., Tran C., Halpern B.S., Stout D., Phelps M.E., Weber W.A. (2006). Impact of animal handling on the results of 18F-FDG PET studies in mice. J. Nucl. Med..

[18] Verger A., Filss C.P., Lohmann P., Stoffels G., Sabel M., Wittsack H.J., Kops E.R., Galldiks N., Fink G.R., Shah N.J. (2017). Comparison of (18) F-FET PET and perfusion-weighted MRI for glioma grading: A hybrid PET/MR study. Eur. J. Nucl. Med. Mol. Imaging.

[19] Hennig J., Nauerth A., Friedburg H. (1986). RARE imaging: A fast imaging method for clinical MR. Magn. Reson. Med..

[20] Bradford M.M. (1976). A rapid and sensitive method for the quantitation of microgram quantities of protein utilizing the principle of protein-dye binding. Anal. Biochem..

